# Multi-source transfer learning for facial emotion recognition using multivariate correlation analysis

**DOI:** 10.1038/s41598-023-48250-x

**Published:** 2023-11-28

**Authors:** Ashwini B, Arka Sarkar, Pruthivi Raj Behera, Jainendra Shukla

**Affiliations:** Human-Machine Interaction Lab, Indraprastha Institute of Information Technology, New Delhi, India

**Keywords:** Image processing, Computational models

## Abstract

Deep learning techniques have proven to be effective in solving the facial emotion recognition (FER) problem. However, it demands a significant amount of supervision data which is often unavailable due to privacy and ethical concerns. In this paper, we present a novel approach for addressing the FER problem using multi-source transfer learning. The proposed method leverages the knowledge from multiple data sources of similar domains to inform the model on a related task. The approach involves the optimization of aggregate multivariate correlation among the source tasks trained on the source dataset, thus controlling the transfer of information to the target task. The hypothesis is validated on benchmark datasets for facial emotion recognition and image classification tasks, and the results demonstrate the effectiveness of the proposed method in capturing the group correlation among features, as well as being robust to negative transfer and performing well in few-shot multi-source adaptation. With respect to the state-of-the-art methods MCW and DECISION, our approach shows an improvement of 7% and $\sim$15% respectively.

## Introduction

Facial expressions play a crucial role in social communication as they are good indicators of the emotional state and intents of humans^1^. Understanding one’s emotional state is pivotal in providing the responses one intends for their actions. With the advances in deep learning and sensor technologies, there has been increased attention to capturing the emotional state of the person from the facial expression^2,3^. Automatic facial emotion recognition has seen wide applications in scenarios where data acquisition opportunities are limited such as among individuals with intellectual disabilities, children with autism, etc.^4^, robotics^5,6^, entertainment^7–9^, assistive systems^10^ and more.

Recent studies have achieved tremendous progress in facial emotion recognition using deep learning techniques in situations where they are furnished with a large amount of annotated data^11,12^. The FER datasets usually have a limited amount of data samples and differ in emotion labels, poses and conditions of data collection, which limit their compatibility with deep learning frameworks. Procuring large amounts of facial expression data is also challenging given the privacy concerns related to the sharing of identifying facial images, time and resource constraints. Moreover, there are pre-trained classifiers available for identifying the emotional labels in these data. Each of these networks has the knowledge gained from the collected set of facial emotion patterns. To address the challenge of data scarcity, recent research has manoeuvred transfer learning techniques to relay the knowledge captured from one domain to another.

With the availability of multiple datasets, Multi-Source Domain Adaptation (MSDA)^13^ has gained interest, wherein multiple labelled source domains are used to transfer the learnt knowledge to the target domain. The generalizability of multi-source transfer learning in providing a broader view of the target domain has been demonstrated in prior works^14–17^. One common approach for multi-source domain adaptation is to align the source and target feature representations and reduce the classification loss on the source data^18,19^. Another approach is to encourage agreement across source target predictions rather than working on the feature representations^20^. Similar to its success in domains like text, we hypothesise that in FER as well, the domain information provided by multiple source tasks can be leveraged in capturing the underlying characteristics of emotion expression in humans across domains irrespective of the variations in poses, cultures, ethnicity and condition mismatch.

All the above methods assume access to the source data for adapting the source knowledge to the target domain. In practical scenarios owing to privacy, security, and management reasons, only a trained source model is available where access to the source data, as well as control over the source training, is restricted. In this work, we explore the multi-source domain adaptation (MSDA) setting where only multiple pre-trained source models are available for supervising the domain adaptation while the source datasets are not accessible. Recent research addresses this by adapting single source^21^ or multiple sources as in DECISION method^22^ to the target domain without access to the source data, meanwhile assuming that sufficient target data is accessible. But in practical scenarios like facial emotion recognition in children with autism, medical data and so on, acquiring sufficient training data is challenging owing to the distinctive nature of the cohort. Inspired by prior work, Maximal Correlation Weighting (MCW)^14^, we assume a few-shot setting where few labelled target samples are available for supervising the adaptation. To summarise, we aim to address the problem of FER by constructing a multi-source domain adaptation problem where the source dataset is unavailable, whereas we have access to a small target dataset with few samples.

To address the above-defined problem, we leverage the maximal correlation approach^14^, where the features generated by the pre-trained networks are represented as maximal correlation functions. We particularly look into the multivariate correlation^23^ of the source features with the target domain, thereby capturing the complex association between the high-dimensional source features and the target. To sum up, the main contributions of this work are:

- We propose a multi-source domain adaptation approach for facial emotion recognition by leveraging the multivariate maximal correlation analysis using a few labelled target samples without access to source data.
- We evaluate our approach on the FER task by conducting extensive experiments on benchmark FER datasets. Experiments show that our approach consistently improves the results over the best single-source model. Further, our approach outperforms state-of-the-art FER - MCW^14^ and DECISION^22^ methods across multiple datasets.
- We also show the ability of the approach to generalize over domains outside FER by performing a general image classification task with the CIFAR-100 dataset.

This paper is organized as follows: In Section "Related work", we present the related work. In Section "Preliminaries", we introduce the preliminary concepts used in the proposed work, and in Section "Proposed model for multi-source transfer learning using multivariate correlation analysis (MSTL-MCA)", we develop the proposed method for multi-source transfer learning. In Section "Experimental setup", we demonstrate the experiment details on the facial expression datasets as well as on standard image datasets and discuss the results in Section "Results and analysis" and Section "Conclusion" summarises the work.

## Related work

Recent research focuses on deep learning techniques for automatic facial emotion recognition. This section discusses various deep learning approaches present in the literature for facial emotion recognition.

### Transfer learning in facial emotion recognition

FER has witnessed a breakthrough with the advent of deep learning techniques, which eliminated the tedious pre-processing phase and provided end-to-end solutions from the input visual information to the emotion recognition. An end-to-end learning framework based on a deep region and multi-label was proposed for the detection of facial action units in^24^. Another approach shows that combining multiple networks shows better performance in automatic facial emotion recognition. In this approach, CNN-LSTM and C3D networks were used in conjunction to simultaneously model video appearances and motions^25,26^ proposed a method that is robust to variations in expression intensity by learning the spatiotemporal feature representations for FER. In^27^, face detection with face alignment deep neural network with inception layers is used to address the FER problem. Research also shows that pre-processing the images before feeding them to deep neural networks improves the classifier performance. Pre-processing image data before being fed into a convolutional neural network (CNN) has shown to have a positive effect on the learning process^28^. In this, rather than feeding raw input, the data were pre-processed to extract expression-specific features from a face image and were then fed into a convolutional neural network for emotion recognition. Convolutional neural network with attention mechanism (ACNN) has been shown efficient in perceiving the occlusion regions of the face and has been used to recognize facial emotions in the wild in the presence of occlusions^29^.

One of the characteristics of these deep learning methods is the need for a large amount of data for training the deep neural network architecture. Training the deep learning framework with the relatively smaller FER datasets leads to over-fitting. Access to such a large collection of data is often challenging, especially in applications involving children. Further, annotating data for FER is an extremely time-consuming and resource-sensitive process.

To mitigate this, there are several studies that propose transfer learning techniques where knowledge gained from models pre-trained on similar large datasets is transferred to the domain-specific learning task. Knyazev et al.^30^ proposed an ensemble of industry-level face recognition networks pre-trained on large facial emotion databases such as FER2013 for emotion recognition. Aly et al.^31^ proposed a multi-stage Progressive Transfer Learning method by fine-tuning the Alexnet convolutional network and demonstrated the FER performance on VT-KFER and 300W datasets. Ngo et al.^32^ demonstrated a transfer learning approach using the SE-Resnet-50 model pre-trained on the VGG-Face2 database along with a novel cluster loss function to transfer the high-level features learned by the network to the FER. These methods leverage a single source transfer learning approach where the source networks are trained on data from a single domain.

### Multi-source domain adaptation

With the availability of a large number of datasets, even though with limited data samples, it is an intuitive step to take advantage of the diverse information comprehended by the different sources. Multi-source transfer learning has been explored widely in text classification^33^, pattern recognition in EEG signals^34^, speech recognition^35^ etc. One of the approaches for multi-source transfer learning relies on the assumption that the target task can be represented as a weighted combination of the source tasks^36^. One common approach to learning these combination weights in multi-source transfer learning is latent space transformation, which learns a common function across the different source tasks by optimizing the overall loss function. Guo et al.^37^ proposed a supervised multi-source domain adaptation method by establishing a set of distance measures to add to the loss function to be minimized for better domain adaptation. Zhao et al.^38^ used ${\mathscr {H}}$-divergence to measure the distance between two domain distributions^13^ employed adversarial methods using GAN loss for generating domain-independent feature representations. Meta-learning models (MAML)^39^ have been developed, which can be used as a starting point for learning a good model fine-tuned to a target task, using only a few local gradient steps. With performance gap as a measure of divergence of source and target distribution and instance weighting, Wang et al.^40^ proposed a boosting approach for transfer learning exploiting the label information in the target domain.

Many of the MSDA approaches train domain-specific classifiers and learn a weighted ensemble of these source classifiers for the target prediction^13,14,41,42^. These methods expect access to the target dataset for learning a rule for combining the source classifiers. Guo et al.^37^ used a point-set distance metric and meta-learning approach to combine the source models for target prediction^41^. Yue et al.^18^ exploited domain-invariant and class discriminative features augmented with alignment loss for MSDA. Ahmed et al.^22^ addressed the MSDA problem without accessing the source data by employing *Information Maximisation (IM)* and pseudo-labeling strategy^22^. Their approach demanded sufficient target data for training the ensemble source network, which may not be practical in many applications. Lee et al.^14^ introduced a multi-source transfer learning method in image classification, which also addresses the data privacy concerns of the transfer learning methods. In this, the knowledge gained by the multiple source networks can be transferred to the target task without access to source samples. Considering the pre-trained source networks as black boxes, they used bivariate maximal correlation analysis to train the ensemble of source networks and a weighted combination of features extracted from the source networks was used to build the target classifier. This approach considers the features independent of each other and leaves out the group correlation among the features within each source while combining the source networks. Recent studies show evidence of better generalization in multi-source transfer learning when compared to single-source one in FER applications^43^.

The review shows that there have been limited studies exploring the possibilities of multi-source transfer learning in automatic FER. These studies either worked on single-source transfer learning for e.g.^44^ or required access to the source datasets for domain adaptation for e.g.^31^. Inspired by the success in multi-source transfer learning in other domains like NLP, for e.g.^45,46^ and considering the challenge of data scarcity in FER, we propose a multi-source transfer learning approach to train a target classifier from a weighted ensemble of pre-trained source networks trained on different source datasets. We utilize the features extracted from different pre-trained source networks and construct a target classifier for the target FER task. Our method for aggregating the features from pre-trained networks relies on the hypothesis that in real-world situations, a feature may exhibit a weak correlation with the target class when considered individually, but when taken into consideration together, they can generate a strong correlation^47^. To address this, we propose the use of multivariate maximal correlation to determine the weights of the source networks that contribute to the target classification task. We leverage the Alternating Conditional Expectation (ACE) based^23^ method, which captures the non-linear association among random variables in a multivariate setting. We further perform few-shot training with target samples for learning the target classifier to demonstrate the effectiveness of our method in scenarios with limited training data.

## Preliminaries

### Multivariate maximal correlation analysis

The maximal correlation was first introduced and developed by Hirschfeld^48^, Gebelein^49^, and Rényi^50^ as a measure for the non-linear association between two random variables $X_1$ and $X_2$. It measures the strength of association among two random variables and characterises the non-linear transformations of the variables. We analyse the multivariate correlation of the features on the target classifiers and build an effective and computationally efficient approach for multi-source transfer learning.

#### Definition 1

(*Maximal Correlation*) Given two jointly distributed random variables $X, Y \in {\mathscr {X}}$ with positive variance, the maximal correlation of (*X*, *Y*) is defined as:

1 $$\begin{aligned} \rho (X;Y)\triangleq (f^*,g^*) \triangleq \mathop {{{\,\mathrm{arg\,max}\,}}}\limits _{ \begin{array}{c} {f: {\mathscr {X}}\rightarrow \mathop {{\mathbb {R}}}, g: {\mathscr {X}} \rightarrow \mathop {{\mathbb {R}}}}\\ {\mathop {{\mathbb {E}}}[f(X)]=\mathop {{\mathbb {E}}}[g(Y)]=0}\\ {\mathop {{\mathbb {E}}}[f^2(X)]=\mathop {{\mathbb {E}}}[g^2(Y)]=1} \end{array} } \mathop {{\mathbb {E}}} \big [f(X)^T g(Y) \big ] \end{aligned}$$

where expectations are with respect to joint distribution $P_{X,Y}$. $(f^*,g^*)$ are referred as maximal correlation functions.

Maximal correlation is equal to the second largest singular value of a scaled joint probability distribution matrix. The singular vectors of the scaled probability distribution matrix could characterize the optimal transformations of the variables when they are discrete^23^. Given $f^* = \{f_1,f_2,...\}$ and $g^* = \{g_1,g_2,...\}$ with the associated singular values $\rho _1,\rho _2,...$ the joint probability distribution $P_{X,Y}$ is given by^51^ :

2 $$\begin{aligned} \frac{P_{X,Y}(x,y)}{P_X(x)P_Y(y)}=\sum _{i=1}^\infty \rho _{i}f_i(x)g_i(y) \end{aligned}$$

and

3 $$\begin{aligned} {P}_{Y|X}(y|x) = P_Y (y) \big ( 1+\sum _{i=1,2,...} \rho _{i}f_i(x)g_i(y)\big ) \end{aligned}$$

In the case of the system of continuous random variables, most of the correlation measurements consider the pairwise relationship between the variables. In real-world datasets, data instances are represented as high dimensional multivariate random variables $(X_1, X_2,...X_d)$. Extending definition 1 to multivariate random variables, maximal correlation among real-valued multivariate random variable $X = \{X_i\}^d_{i=1}$ can be given as

4 $$\begin{aligned} \rho ^* (X_1, X_2,...X_d):= \max _{f_1,f_2...f_d} \rho (f_1(X_1),f_2(X_2),...f_d(X_d)) \end{aligned}$$

Using bivariate measures to capture the multivariate relationships may not be efficient in capturing the association among the variables^52^. Methods like Maximal Information Coefficient (MIC)^53^ and Canonical Correlation Analysis (CCA)^54^ consider either two dimensions or linear correlations. In real-world scenarios, a feature may correlate weakly with the target class if considered individually, but when considered as a group, it can lead to a strong correlation^47^. Further, it is computationally expensive to evaluate all the pair-wise relations. Thus, the computation of maximal correlation in multivariate data eventually turns into an optimization problem with complexity quadratic to the dimension, i.e. $O(n^2)$ where *n* is the feature dimension. By the above approach, for a *n* dimensional data to find the correlation among the random elements, each $X_i$ is paired with $n-1$ other elements, and solving the maximal correlation means optimizing these $n(n-1)/2$ transformation functions. Multivariate maximal correlation analysis solves this by considering the group correlation among the features^52^. Maximal correlation eliminates the assumptions on data distribution and captures non-linear relations.

Based on Alternating Conditional Expectation (ACE)^23^, proposed a computationally efficient method for addressing multivariate maximal correlation. It determines a single transformation function corresponding to each random variable, thereby reducing the computational complexity of computing multivariate maximal correlation. This approach maximizes the aggregate inner products between transformed variables to optimize the correlation functions. Given a system of continuous random variables, this approach infers non-linear transformation functions assigned to each variable represented as vertices of a graph such that the aggregate pairwise correlations over the graph *G* are maximized. The ACE-based approach for computing multivariate maximal correlation is given in Algorithm 1.

**Algorithm 1:**
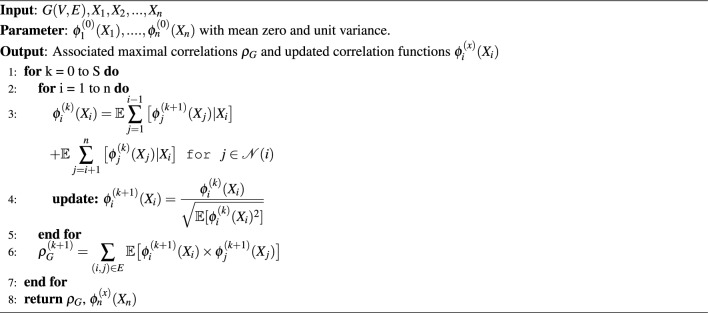
ACE Algorithm to Compute Multivariate Maximal Correlation.

#### Definition 2

Let $G = (V,E)$ be a graph with vertices $V = \{1,2,....n\}$ and edges $E \subseteq \{(i,i'):i,i' \in V,i \ne i'\}.$ The multivariate maximal correlation of $(X_1, X_2...X_n)$ given G is

5 $$\begin{aligned} \rho _G (X_1,X_2,...X_n):= \sup _{(f_1,f_2,...f_n)} \sum _{(i,i')\in E} \mathop {{\mathbb {E}}}[f_i(X_i),f_{i'}(X_{i'})] \end{aligned}$$

such that $$\mathop {{\mathbb {E}}}[f_i(X_i)]=0, \text {and} \mathop {{\mathbb {E}}}[f_i(X_i)^2]=1, \forall 1 \le i \le n$$

## Proposed model for multi-source transfer learning using multivariate correlation analysis (MSTL-MCA)

**Figure 1:**
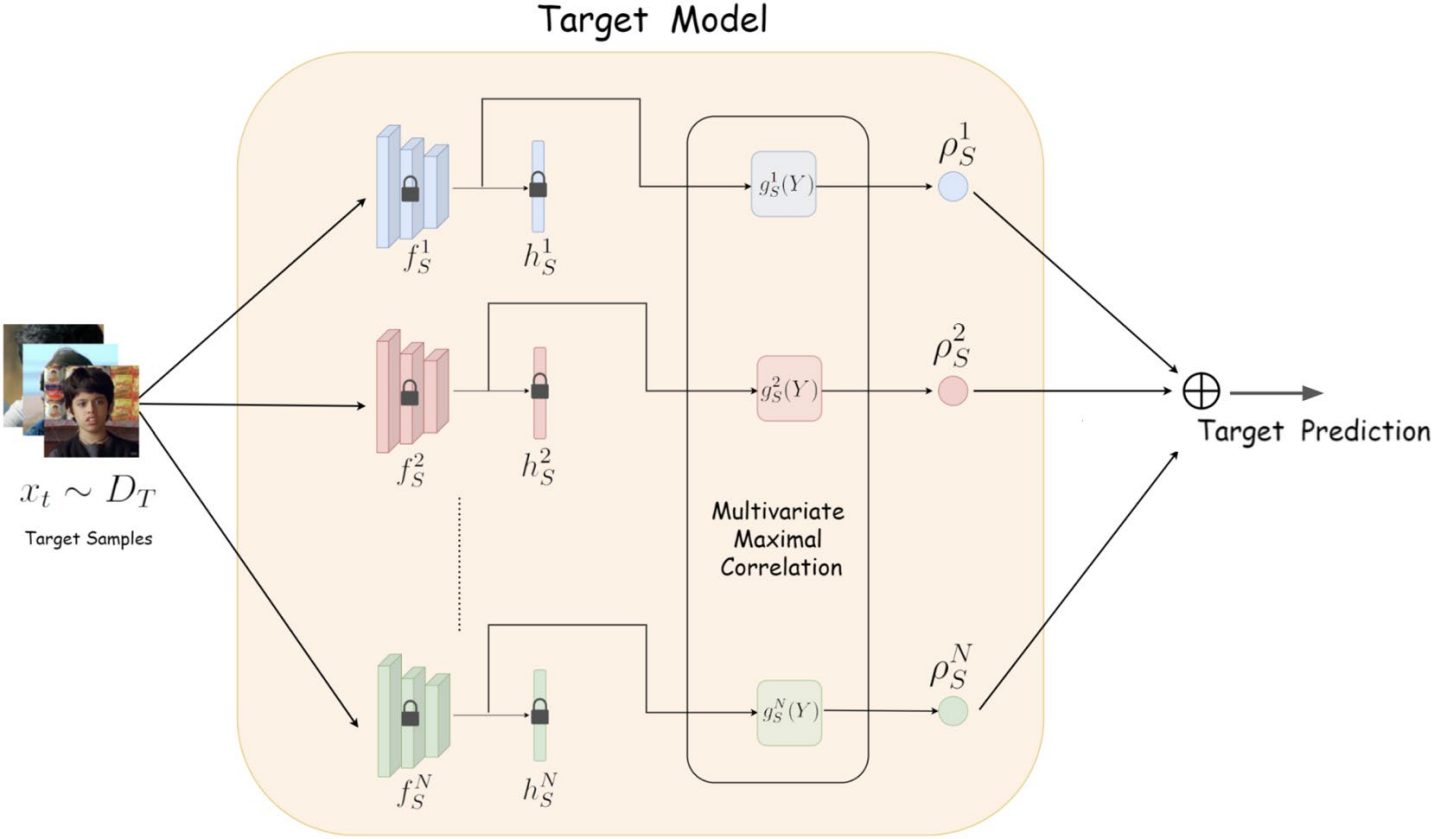
Proposed model architecture for MSTL-MCA.

**Problem Setting**: We formulate the facial emotion recognition with scarce data as a multi-source domain adaptation (MSDA) problem, in which there are *N* labelled source domains and one target domain with few labelled samples. Let the input space be ${\mathscr {X}}$, and the classification is among *M* categories. We represent the pre-trained source models as $\{\theta _S^j\}_{j=1}^N$ where the $j^{th}$ model is represented as $\{\theta _S^j\}: {\mathscr {X}}\rightarrow \mathop {{\mathbb {R}}}^{M}$ is a classifier learnt from source dataset $D_S^j = \{x_{S_j}^i,y_{S_j}^i\}_{i=1}^{N_k}$, with $N_k$ data points. $x_{S_j}^i$ denotes the $i^{th}$ source image in source $S_j$ and $y_{S_j}^i$ denotes the corresponding label. Given a target dataset $D_T = \{x_T^i,y_{T}^i\}_{i=1}^{N_T}$, with few samples, the problem we are addressing is to learn a classifier $\{\theta _T\}:{\mathscr {X}}\rightarrow \mathop {{\mathbb {R}}}^{M}$ using the ensemble of pre-trained source classifiers without access to source datasets. The data points are facial expression images represented by $(x_1,y_1),...(x_n,y_n)$ where $(x,y)\in {\mathscr {X}}\times \{1,2...M\},$ the feature $x \in \mathop {{\mathbb {R}}}^d$ is sampled from input space ${\mathscr {X}}$ and label $y \in \{1,2,..M\}$. In the absence of source training data, we leverage the knowledge learned by *N* pre-trained networks trained on similar but different source datasets and learn the classifier $\theta _T$, which has a low classification error on the target dataset. The high-level overview of the proposed architecture is given in Fig. 1.

We represent each source model $\{\theta _S^i\}$ as the composition of two transformations :

1. The mapping $f_S^i$ transforming the input vector into feature vector of length $d_i$, $f_S^i: {\mathscr {X}}\rightarrow \mathop {{\mathbb {R}}}^{d_i}$ where $d_i$ is the length of the feature vector of source *i*
2. A classifier $h_S^i: \mathop {{\mathbb {R}}}^{d_i}\rightarrow \mathop {{\mathbb {R}}}^M$ from the feature vector into the output label, $Y^{s_i}$. This forms the hypothesis function.

Thus $$\theta _S^i = (f_S^i \circ h_S^i)$$

For the sake of better explainability, we have considered the feature length to be the same for all the source tasks and $d_i=d_j,\forall i,j =1,2,...N$. To build the target classifier, given N source tasks, with feature functions $\{f_S^i\}_{i=1}^N$, we optimize respective $\{g_S^i\}_{i=1}^N$ which is the hypothesis function such that the aggregate maximal correlation of functions $f_S^i$ and $g_S^i$ given by

6 $$\begin{aligned} \rho ^* = \sum _{i=1,2,...N} \mathop {{\mathbb {E}}} {}_{{\hat{P}}_{X,Y}^t}{f_S^i}(X)g^i_S(Y)] \end{aligned}$$

where ${{\hat{P}}_{X,Y}^T}$ is the empirical joint distribution of the target data.

For each source, the optimal correlation function, $g_S^i$ corresponding to feature function $f_S^i$ and the corresponding correlation coefficient could be computed^14^ as

7 $$\begin{aligned} g_S^i(Y)= & {} \mathop {{\mathbb {E}}} {}_{{\hat{P}}_{X,Y}^T}[f_S^i(X)] \end{aligned}$$

8 $$\begin{aligned} \rho {(f,g)}= & {} \mathop {{\mathbb {E}} {}_{{\hat{P}}_{X,Y}^T} [f_S^i(X)g_S^i}(Y)] \end{aligned}$$

While considering the high dimensional image data, it is interesting to analyze the group correlation of the multivariate data rather than the binary correlation among the individual features, $f_S^j(X)$ and $g_S^j(Y)$. Multivariate correlation analysis may reveal hidden complex interactions affecting the classification task^23^. Hence, we leverage the multivariate correlation among the group of features extracted by the feature extraction layer to compute the function $g_S^j(Y)$. In this direction, we apply network maximal correlation, an ACE-based multivariate maximal correlation approach given in definition 2, which characterizes the multivariate non-linear association between random variables.

We train the ensemble of the source classifiers on target samples to optimize $g_S^i$ to maximize the aggregate maximal correlation given in equation 6 i.e.

9 $$\begin{aligned} g^i_S= \underset{{{\tilde{g}}^{i_S}}}{\textrm{argmax}} \; \rho * \end{aligned}$$

The correlation value for each pair of $(f_S^i,g_S^i)$ gives the strength of association between the functions. Since we are considering the group correlation of features with the target, the $\rho ^i_S$ represents the combined weighted contribution of the feature functions of each source network to the ensemble classifier for the target domain.

10 $$\begin{aligned} \rho ^i_S = \mathop {{\mathbb {E}}} {}_{{\hat{P}}_{X,Y}^t}[f_S^i(x)g^i_S(y)] \end{aligned}$$

Finally, the prediction of the target label on the test data is given by

11 $$\begin{aligned} {\hat{y}} = \underset{y}{\textrm{argmax}} {\hat{P}}_{Y|X}(y|x), \end{aligned}$$

where

12 $$\begin{aligned} \underset{y}{\textrm{argmax}} {\hat{P}}_{Y|X}(y|x) = {\hat{P}}_Y^t\big ( 1+\sum _{\begin{array}{c} i=1,2,...N \\ j=1,2,...l_i \end{array}} \rho ^{i}_Sf_S^i(x)g_S^i(y)\big ) \end{aligned}$$

The procedure for the NMC-based multi-source learning is given in Algorithm 2.

**Algorithm 2:**
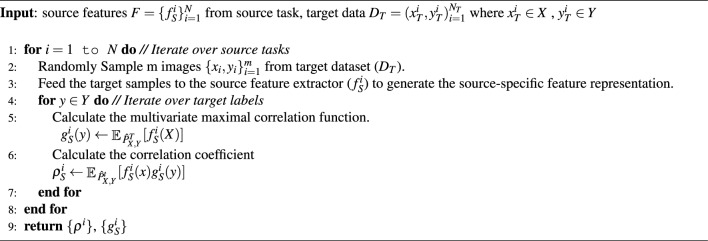
Proposed MSTL-MCA approach.

## Experimental setup

### Task and datasets

*Facial Emotion Recognition* To understand the performance of our approach, we designed a set of experiments on the FER task using four FER datasets: FER 2013, RAF-DB, JAFFE, and CAFE under different source-target settings. Further, we investigated the efficiency of the approach on a novel FER dataset, the Child Facial Expression Dataset (CFED), curated by the authors. The dataset details are given below:

- FER2013 dataset^55^ The 2013 Facial Expression Recognition dataset (FER2013) is a dataset provided by Kaggle, introduced at the International Conference on Machine Learning (ICML) in 2013^56^. The dataset contains 35887 images, and each image has been categorized into 7 different types of emotion categories. The images in the dataset are registered hence the face appears in the center of the image dataset.
- JAFFE dataset^57^: The Japanese Female Facial Expression (JAFFE) dataset consists of 213 images of different facial expressions from 10 different Japanese female subjects.
- RAF-DB dataset^58^: The RAF-DB dataset has 29672 real-world images labelled with 7 basic emotions and 12 compound emotions.
- CAFE dataset^59^ The CAFE set features the facial expression data of a racially and ethnically diverse group of 2- to 8-year-old children posing for six emotional facial expressions and neutral emotion. The CAFE dataset consists of facial expression data of 90 female and 64 male children from varying ethnicities.
- CFED dataset: The Child Facial Emotion Dataset (CFED) was collected, annotated, and prepared by our research group. There are limited annotated facial datasets for child facial emotion expression especially in the global south where active research in child emotion recognition is limited. The CFED dataset was collected by video search on child videos from YouTube under the Creative Common Licence, which allows the use of the videos for research. The manually retrieved video frames with expressed emotions were annotated by the research team. It consists of 606 images of children from Indian ethnicity representing 6 emotion classes - Anger, Fear, Happy, Neutral, Sadness, and Surprise.

**Figure 2:**
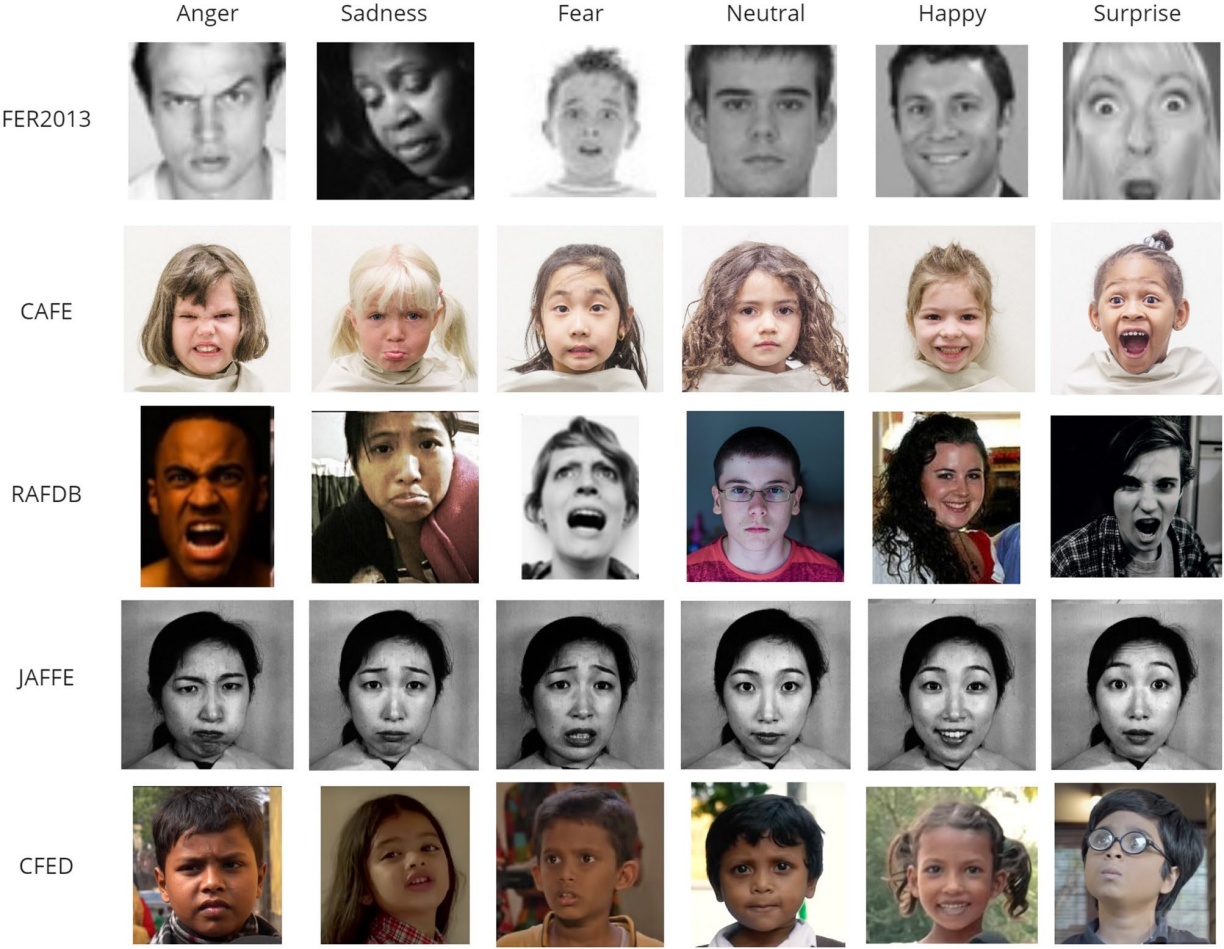
Sample images for FER datasets.

For our experiments, we used the six emotional classes - Anger, Fear, Happy, Neutral, Sadness and Surprise from the FER datasets: FER 2013 (F), RAF-DB (R), JAFFE (J), and CAFE (C). Each domain has 600 labelled samples for training, i.e. 100 from each class label, and the testing set has 60 samples, i.e. 10 from each class label. Samples from each FER dataset are represented in Fig. 2

*Image classification* We further considered the image classification to demonstrate the generalizability of the approach. For this, we conducted experiments on the benchmark image dataset CIFAR-100. We followed the specific experiment setting proposed by Lee et al.^14^.

- CIFAR 100: The CIFAR-100 dataset has 100 classes containing 600 images each. There are 500 training images and 100 testing images per class. For our experiment, we have considered ten different source tasks, each consisting of 2 non-overlapping classes. All images were resized to 32x32, and the pixel values were normalized to zero mean and unit variance.

**Figure 3:**
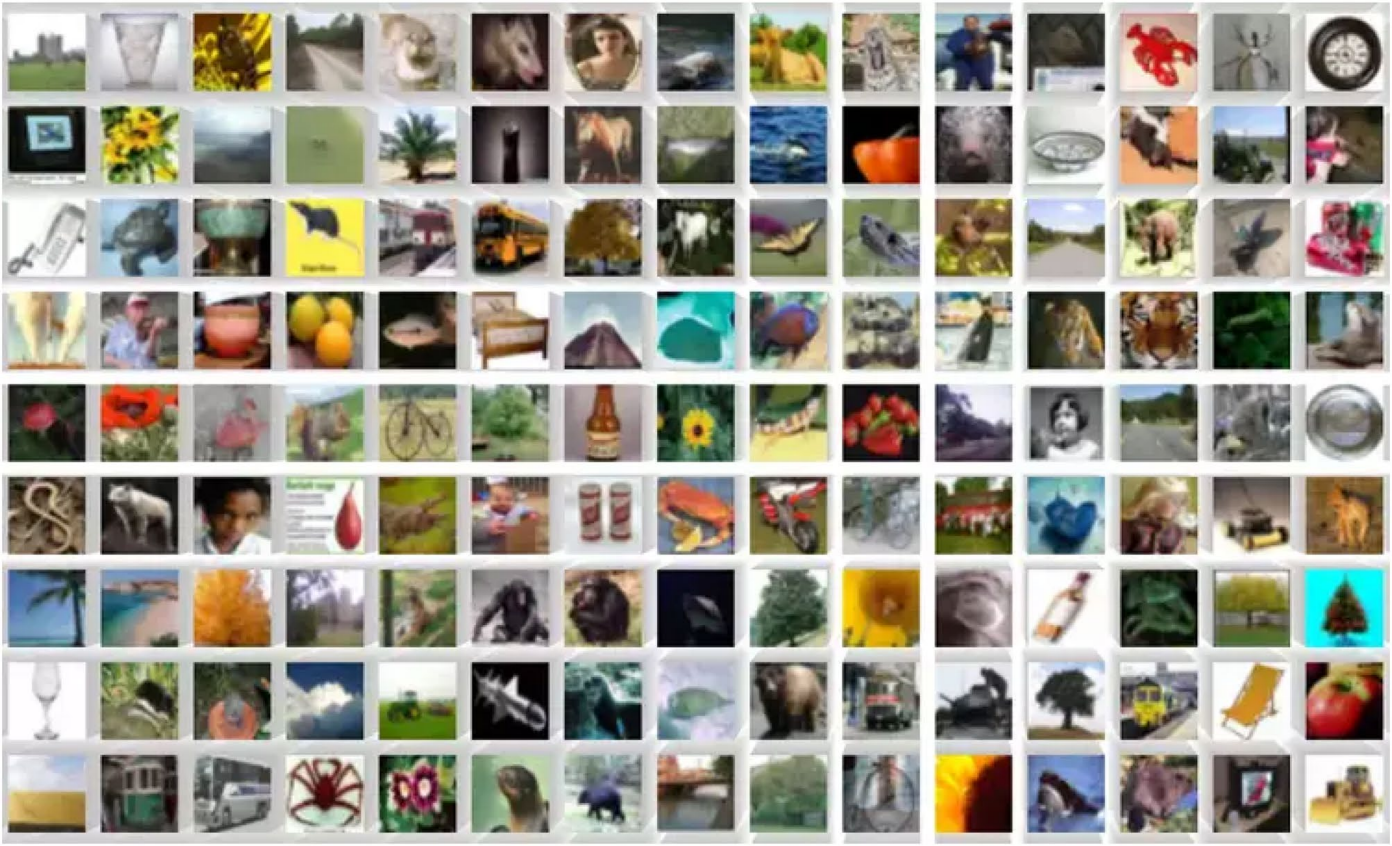
Sample images for the CIFAR-100.

For our experiments, we randomly selected 10 non-overlapping class categories from the source task. For training, each source dataset had 500-labeled samples per class. Samples from the CIFAR-100 dataset are represented in Fig. 3.

### Experiments

We investigated the performance of the MSTL-MCA approach on multiple FER datasets. In this, we compare our approach against two different baselines, which are commonly followed in the literature. The first one is the best single-source adaptation among the other sources (best-SS)^37^, and the other is a unified multi-source model (uni-MS) where all the source data are combined to form a single source and single-source domain adaptation is performed on the target data^60^. There is very limited work in multi-source domain adaptation without access to the source data. We compared our approach with Lee et al.^14^, where they have considered feature independence while performing maximal correlation-based source weighting for multi-source domain adaptation. Further comparison was made with the DECISION^22^ where unsupervised multi-source domain adaptation is being addressed using Information Maximisation loss and clustering-based pseudo labelling. We conduct experiments by selecting the target dataset in a round-robin fashion from among the set of FER datasets and keeping all the other datasets as the source datasets. We extended our experiments by evaluating the method on a novel CFED dataset as the target and the other standard datasets as the source task.

We further conducted experiments on image classification with the CIFAR-100 dataset. We followed the same experiment setting^14^ for the comparison. For our experiment, we have considered ten different binary classification tasks as sources, each consisting of 2 non-overlapping classes. The source tasks were trained with 500 samples to generate the source network weights.

### Implementation details

*Pre-training* In our experiment on FER, we constructed 6-way (anger, sad, happy, surprise, neutral, fear) emotion classification on different FER datasets as the source tasks. The disgust class was discarded as it was not present in all the FER datasets considered. It is important to note that the source data samples were used only for pre-training the source tasks and not for training the target classifier. In other words, the source data samples were used to create a foundation or base knowledge for the source tasks but not for directly training the target classifier. This distinction is important because it highlights the importance of separating the pre-training and training stages and the potential benefits of using pre-trained networks for feature extraction. In real-world scenarios, the assumption is that these pre-trained source networks are available for feature extraction but are not trainable. Similarly, for the image classification task, we constructed binary classification tasks CIFAR-100 classes. We selected 10 non-overlapping pairs of classes from CIFAR-100 for classification in the source tasks.

All images were resized to 32x32, and the pixel values were normalized to zero mean and unit variance. We used ResNet18 architecture^61^ similar to^21,22^ for pre-training the source tasks with parameters tuned for the specific dataset considered. We repeated all the experiments with LeNet architecture as well, which is a simple convolutional neural network architecture. The Cross-Entropy Loss was used as the loss function, and the Stochastic gradient descent (SGD) optimizer was used with the learning rate equal to 0.001, momentum set to 0.9, and the number of epochs to 100. These networks were considered as the black box pre-trained networks for the multivariate correlation analysis, where the features from the pre-trained networks will be extracted and further trained on target samples to compute correlation functions and coefficients for each set of features extracted from the pre-trained source networks, which will be used for the classification of the target test samples. Here, the black box implies that we do not have control over the training of the source networks but can only generate features pre-trained on these networks.

*Training* Once the pre-trained networks are available, the ensemble network is trained with 1, 5, 10, and 20 samples from the target task, during which the multivariate correlation functions and the correlation coefficient are computed. The training was done on Ubuntu Server 20.04 LTS, and the GPU used for training was Nvidia’s RTX 3090. We used the PyTorch framework for all our implementations. To ensure reproducibility and to support open source, the code and the CFED dataset will be made available on request.

**Table 1:** MSTL-MCA Results on different FER datasets RAF-DB (R), FER2013 (F), CAFE (C), JAFFE (J) with different source (s) - target (t) settings.

Setting	s(R+C+J) - t(F)	s(F+C+R) - t(J)	s(F+C+J) - t(R)	s(F+J+R) - t(C)	Average
Random	16.67%	$16.67\%$	$16.67\%$	$16.67\%$	16.67%
uni-MS	23%	$26\%^*$	$19\%^*$	$23\%^*$	22.75%
best-SS	22%	34%	20%	27%	25.75%
DECISION (Ahmed et al. 2021)	26.43%	33%	22.14%	24.07%	26.4%
MCW (Lee et al. 2019)	33%	43%	31.67%	31.67%	34.83%
MSTL-MCA (LeNet)	38%	43%	38.33%	33%	38.08%
MSTL-MCA (ResNet-18)	38.33%	43.33%	35%	35%	37.92%

*indicates instances of *negative transfer*.

## Results and analysis

### Facial emotion recognition

Our approach focuses on multi-source domain adaptation without the need for source data for domain adaptation while also addressing the challenge of limited target data, where only a small number of target samples are available for training. It should be emphasized that in this method, the source data is utilized solely for pre-training the source models. Most recent studies in multi-source domain adaptation, to the best of our knowledge, require labeled data from both source and target domains, as well as a mechanism for learning domain-invariant representations. For a fair evaluation, we compared our method with MCW^14^, which is similar to our approach, which addresses source-free multi-source domain adaptation. Additionally, we compared our supervised approach with the DECISION^22^ algorithm, which also tackles the problem of multi-source domain adaptation, even though it is an unsupervised approach.

We report our results on FER datasets in Table 1. We observe that our method consistently performs better across the different dataset settings and tasks. We observe a mean improvement of $\sim 12\%$ with respect to the best single source performance and compared to the uni-MS, our method gives $\sim 15\%$ improvement in performance (Table 1 in *Average* column). Further, in cases of negative transfer, indicated as ($^*$), our approach is performing better, indicating that it is robust to negative transfer. Negative transfer happens when transferring knowledge from a less related source, which may inversely affect the target performance. It is shown in cases where the best single-source model outperforms the unified multi-source model, indicating the adverse effect from unrelated sources. Compared to the MCW method, MSTL-MCA gives an improvement of 3.74% improvement. This signifies that group correlation among the features is capable of capturing the differentiating features in multi-source adaptation, and hence, the classification accuracy is higher. Even though an unsupervised algorithm, the DECISION approach addresses multi-source adaptation with similar settings. We compared our results with DECISION and obtained an improved performance of $\sim {11}$%.

Further, even with the newly curated CFED dataset, our proposed approach confirms its efficiency with similar trends in performance. The results for the CFED dataset are given in Table 2. The results reported are for 20 shots. With respect to the best-performing model, i.e. the MCW, it shows an improvement of 7% and $\sim 15\%$ with DECISION. We have run the experiments for different shots, and the results are given in Table 3. The results show that the proposed method performs better in few-shot settings. This analysis illustrates that our algorithm’s performance significantly improves up to 20 shots, after which it gradually converges. At this point, the model with joint training approach has received a sufficient number of samples to learn their parameters and the addition of more samples no longer yields significant knowledge gains.

**Table 2:** MSTL-MCA results on CFED dataset.

Model	Accuracy
uni-MS	19.00%
best-SS	17.00%
DECISION (Ahmed et al. 2021)	30.02%
MCW (Lee et al. 2019)	38.00%
MSTL-MCA (LeNet)	42.00 %
MSTL-MCA (ResNet-18)	45.00%

**Table 3:** MSTL-MCA elbow point analysis for CFED dataset with source as (F+R+C+J) and target as CFED.

Dataset	1-shot	5-shot	10-shot	20-shot	25-shot	30-shot	60-shot
CFED	40%	40%	43%	**45**%	45.67%	45.01%	45.33%

Significant values are in bold.

**Figure 4:**
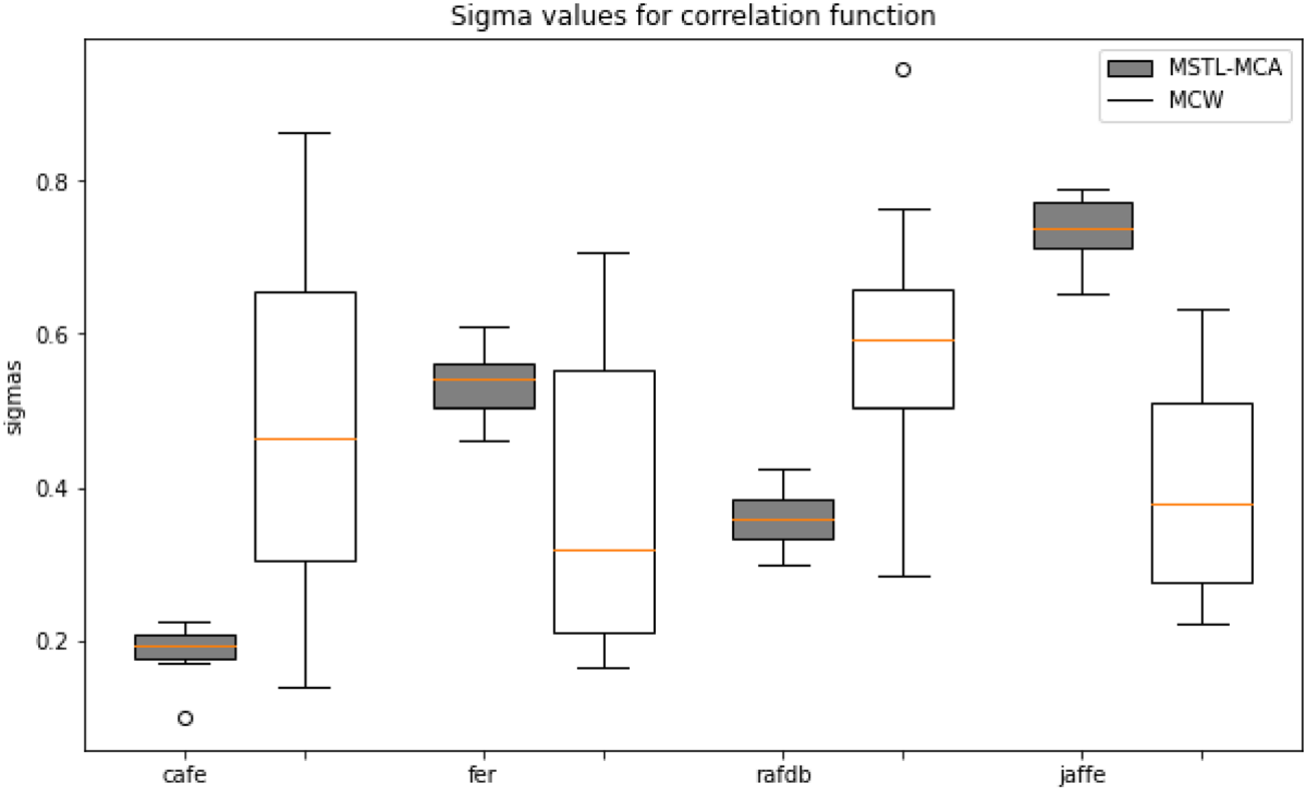
Maximal correlation analysis for CAFE, JAFFE, RAF-DB, FER-2013 as source and CFED as the target over 20 runs.

*Maximum correlation analysis* To study the effect of multivariate maximal correlation in regulating the flow of knowledge from the source to the target task, we conducted the correlation analysis between source and target pairs. For this, we considered CAFE, FER-2013, RAF-DB, and JAFFE as the source datasets and CFED as the target dataset. We computed the correlation coefficient corresponding to each source task for 20 runs. We then compared it with the correlation weighting of the sources computed by MCW^14^ under the same settings. The correlation coefficients for the different source tasks using MSTL-MCA and MCW are given in Fig. 4. The results show that the correlation weighting of our approach for each source is clustered closely around the median when compared to the MCW method, where the weights learned are more variable to the input samples under consideration. This shows that our approach could produce more reliable and accountable results by consistently focusing on the relevant source knowledge over different runs. This accounts for the ability of the model to produce better results than state-of-the-art methods, as seen in Table 2.

For further analysis, we removed the source task with the highest correlation value given by our algorithm, i.e. JAFFE (J), and computed the accuracy for the adaptation task. We observed that average accuracy dropped to 41.99% with a relative drop of $\sim 7\%$. Likewise, removing the task with the lowest weightage given by our algorithm which is CAFE (C), and keeping the other tasks dropped to 44.23% with a relative drop of $\sim 2\%$. With this, we can infer that removing the highly correlated sources leads to a significant drop in accuracy, showing that the source task with high correlation contributes higher to the target classifier learning. Similarly, we compared the effect of multi-variate correlation in the classification task. We compared the correlation strength of our proposed method with the MCW^14^ approach, where binary correlation weighting has been used. The results in Table 2 show that multi-variate group correlation could capture the relevant source knowledge in a consistent and reliable way eventually leading to better performance.

*Statistical Analysis* To further validate our results, we perform statistical analysis. For the null hypothesis, we assume that our proposed model works similar to other algorithms and consider the average accuracy for all the algorithms. We tried 20 different samples for all classifiers on the CAFE dataset and then performed the Kruskal-Wallis H-test (also called one-way ANOVA test on ranks) and the Friedman test. We found the Kruskal-Wallis *H* statistic equal to 65.38, which shows significant statistical importance and outputs a very small $p = 9.33e-13$. Similarly, for the Friedman test, we got a statistical value of 72.37 and $p = 3.28e-14$. As the *p*-value is very small in both the tests and $p<0.05$, we can safely reject the null hypothesis. Hence, we can infer that the performances of all algorithms are not equivalent.

**Table 4:** Average ranks for different methods in post-hoc tests.

Method	Average rank
UNI-MS	5.75
BEST-SS	4.75
DECISION (Ahmed et al. 2021)	4.5
MCW (Lee at al. 2019)	2.875
MSTL-MCA (LeNet)	1.875
MSTL-MCA (ResNet -18)	1.25

Considering that the null hypothesis was rejected, we have two scenarios for a post-hoc test^62^: (1) We perform the Nemenyi post-hoc to compare all algorithms with each other. (2) We perform the Bonferroni-Dunn post-hoc test to compare all the algorithms with a control algorithm (i.e., the proposed method). Both the posthoc tests are performed with alpha values 0.05 and 0.1 as suggested by^62^.

To perform both the post-hoc tests, we calculated the average rank of each algorithm, as shown in Table 4. Average rank (or fractional rank) denotes the algorithm’s performance, i.e. a lower-ranked algorithm performs much better than a higher-ranked algorithm. It is calculated by taking the mean of ordinal ranking, which is done by the simple ordering of the accuracies of respective algorithms. The results given in Table 4 show that our proposed method has a lower rank than other methods and hence outperforms others.

**Figure 5:**
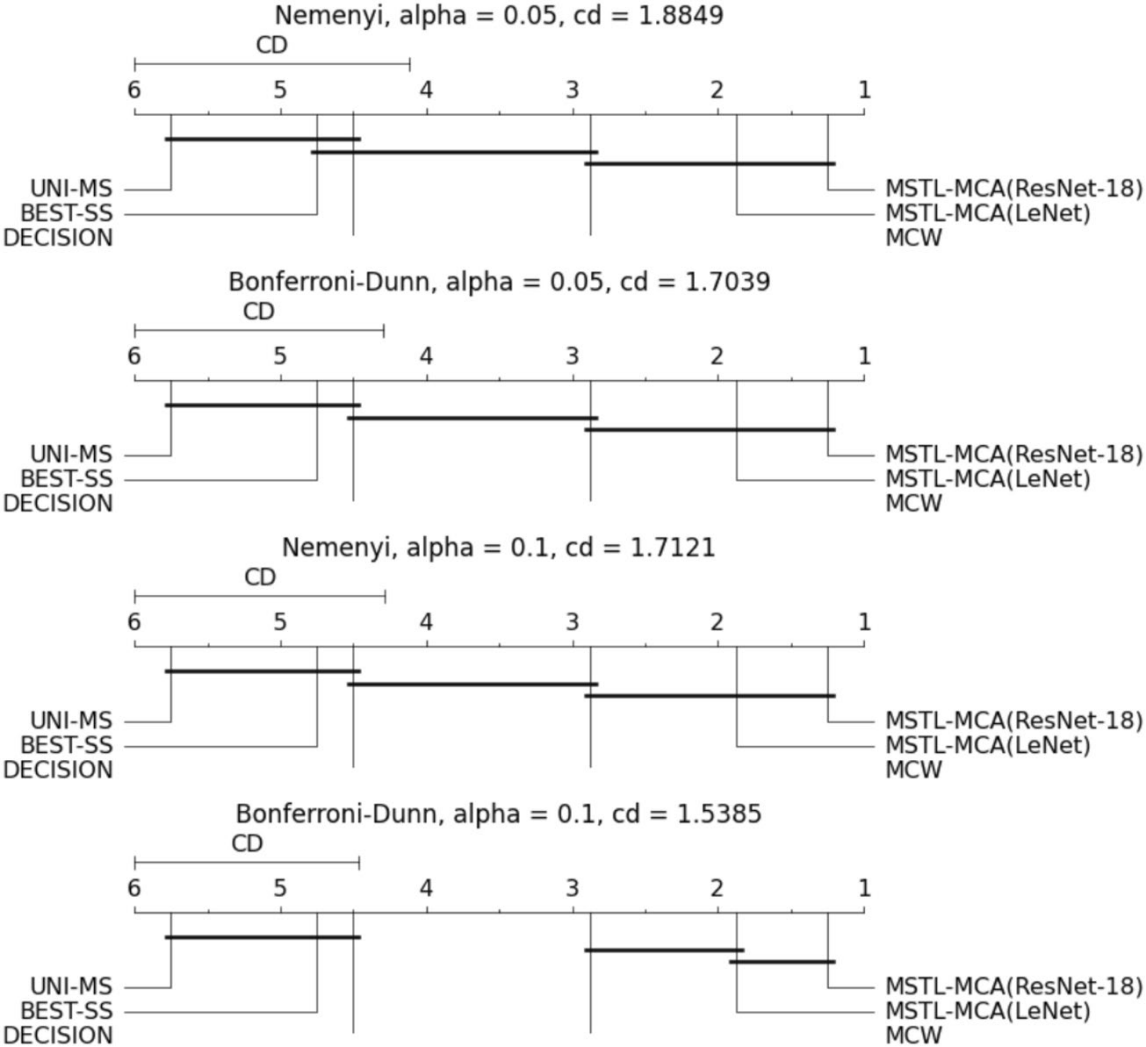
CD Diagram for Nemenyi and Bonferroni-Dunn test. The bold line represents the closely grouped algorithms together.

Then, we compute the critical differences (CD) as per Nemenyi and Bonferroni-Dunn tests plotted in Fig. 5. In the CD diagram, closely performing algorithms are grouped into a single group. Figure 5 shows the graphical representation of the classification accuracies for our problem on the six different methods. In the CD diagram, the lowest (best) ranked algorithms are on the right side of the graph. Hence, the results reveal that UNI-MS, BEST-SS, and DECISION^22^ perform significantly worse than MSTL-MCA (proposed method) and MCW^14^. Further, it can be observed that MSTL-MCA (for both LeNet and ResNet-18) have the lowest ranks among all. This implies that the MSTL-MCA outperforms the other approaches.

**Figure 6:**
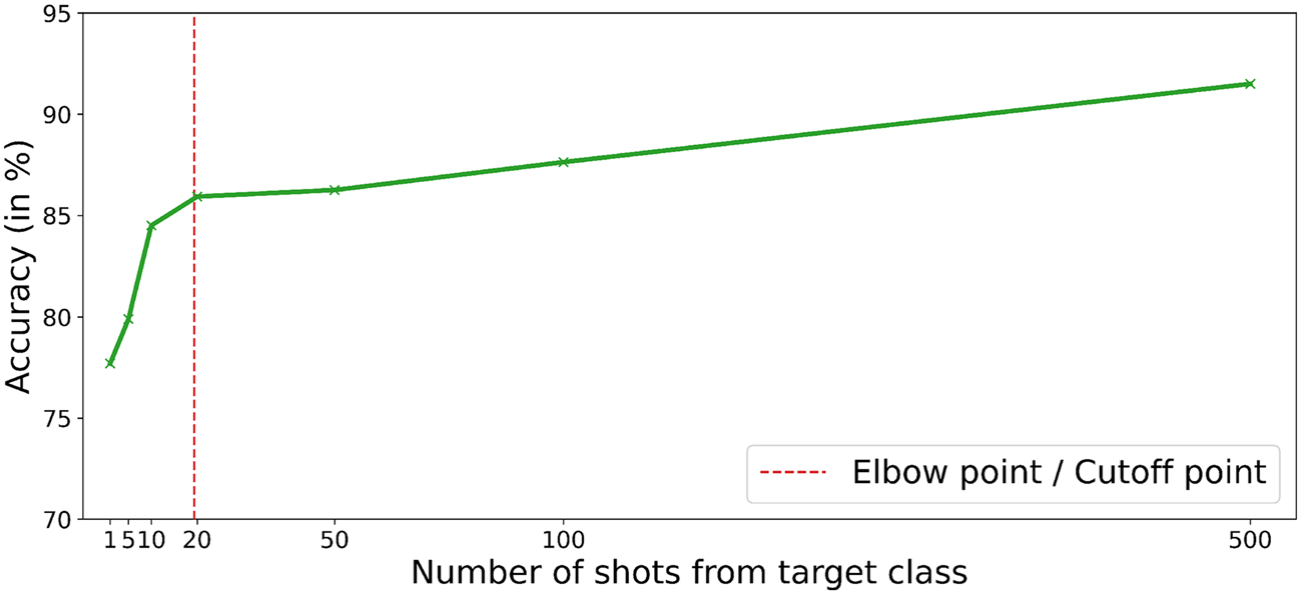
Plot for Accuracy v/s Number of shots for CIFAR-100. The orange line represents the Elbow point.

### Image classification

The results of multi-source adaptation on image classification in the CIFAR-100 dataset are given in Table 5. We could see a similar performance of our method on the image classification task as in the FER task. Our method performs better with an improvement of $\sim 6\%$ in comparison with the state-of-the-art method MCW. It further shows comparable results with DECISION.

**Table 5:** MSTL-MCA results on CIFAR-100 for 10-shots.

Methods	CIFAR- 100
best-SS	60.00%
MCW^14^	78.10%
DECISION^22^	79.50%
MSTL-MCA (LeNet)	83.50%
MSTL-MCA (ResNet-18)	84.53%

*Elbow point analysis* We performed the elbow point analysis on the CIFAR 100 dataset to find the optimal k-value for the k-shot learning approach we used. We can observe from Fig. 6 that in the CIFAR-100 dataset, after 20 shots, the rate of growth in the accuracy is significantly lower concerning shots. So, we can deduce that the elbow point or the knee of the curve is at 20 shots for the CIFAR dataset, and even with a smaller number of samples the algorithm is capable of training the classifier. This shows that our approach has utility in applications, including FER, where there is an unavailability of huge training datasets.

**Figure 7:**
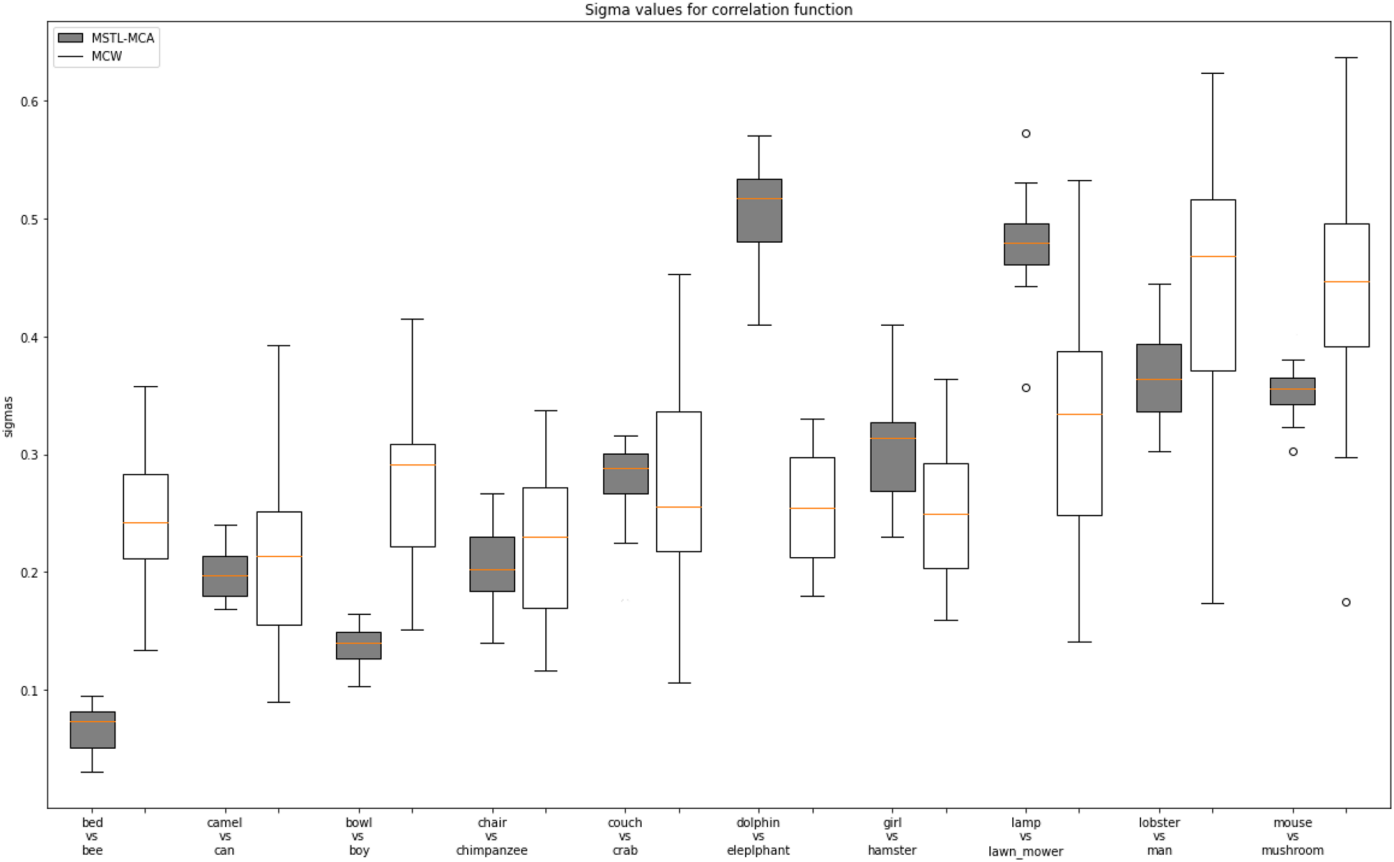
Maximal correlation analysis for CIFAR-100 dataset for 5-shots over 20 runs.

*Maximum correlation analysis* We conducted maximal correlation analysis on the CIFAR-100 dataset with the same settings given in Section "Experimental setup". The weights for the source tasks for the CIFAR-100 dataset are given in Fig. 7. Similar to the FER task, we can see that the correlation weighting of our approach is consistent across the different runs, as represented by the lower spread of the weights.

## Conclusion

In this work, we proposed a multi-source transfer learning approach by leveraging the multi-variate maximal correlation of features extracted from an ensemble of source networks to build a target classifier with unseen classes. We measure the multivariate non-linear association among the features of the source networks using Network Maximal Correlation and optimize the aggregate multivariate maximal correlation over the source tasks to learn the target classifier. The results show that capturing the group correlation of the features with output, as proposed, significantly improves the learning of the target classifier.

We demonstrated the efficacy of our approach in facial emotion recognition using benchmark datasets. We verified and confirmed the performance on the novel CFED dataset with images from YouTube. We investigated the performance of the proposed method in the cross-cultural target classification task by considering the different FER datasets as the source dataset and our novel CFED dataset consisting of facial emotion data of children of Indian ethnicity and having limited samples. We then performed an image classification task using a standard image dataset, the CIFAR-100. We have also shown that the proposed method convincingly performs well even in smaller target datasets with our experiments of k-shot learning with k less than ten shots.

The proposed method enables combining the knowledge from the multiple source networks in an effective and computationally efficient manner and can be leveraged where training data is limited. Further, since the knowledge gained by the source classifier is leveraged to build the target classifier without direct access to the input data in this approach, it ensures improved data privacy which is primal in facial emotion expression data. The proposed method can be generalized to other domains as well while applying transfer learning. The performance of the approach with heterogeneous source tasks with multimodal information can be explored in future work.

**Table 6:** Datasets Used.

Dataset	Source
FER 2013	http://https//www.kaggle.com/datasets/msambare/fer2013
JAFFE	http://https//zenodo.org/record/3451524
RAF-DB	http://www.whdeng.cn/raf/model1.html#dataset
CAFE	https://nyu.databrary.org/volume/30
CIFAR-100	https://www.cs.toronto.edu/%7ekriz/cifar.html

## Data Availability

The image data sets used are available in the public domain and are available upon request, except for the CFED dataset. Owing to privacy concerns, facial expression data (CFED) cannot be made publicly available. However, to ensure the transparency and reproducibility of the research, interested researchers may contact the corresponding author (jainendra@iiitd.ac.in) to discuss potential access to a sanitized version of the dataset in compliance with the applicable confidentiality regulations and ethics requirements. The links to access the public datasets are given in Table 6.
